# Brazeability Study of an Additively Manufactured CuCrZr Alloy to Tungsten Using Various Cu-Based Fillers

**DOI:** 10.3390/ma18245577

**Published:** 2025-12-11

**Authors:** Ignacio Izaguirre, Javier de Prado, Nerea Ordás, María Sánchez, Alejandro Ureña

**Affiliations:** 1 Materials Science and Engineering Area, ESCET, Rey Juan Carlos University, C/Tulipán s/n, 28933 Móstoles, Spain; 2 Instituto de Investigación de Tecnologías para la Sostenibilidad, Universidad Rey Juan Carlos, C/Tulipán s/n, 28933 Móstoles, Spain; 3 Ceit-BRTA and Tecnun, University of Navarra, Paseo de Manuel Lardizabal 15, 20018 San Sebastian, Spain

**Keywords:** fusion reactor, CuCrZr, additive manufacturing, brazing

## Abstract

**Highlights:**

**What are the main findings?**

- Cu13Ge and Cu19Ge fillers produced high-quality joints between CuCrZr and tungsten, while Cu33Ge led to brittle and discontinuous interfaces.
- The Cu20Ti filler generated Ti-rich brittle phases that caused cracking, making it unsuitable for this application.
- CuCrZr softened during brazing due to dissolution of strengthening precipitates, but its hardness could be restored through post-brazing heat treatments; the best result was obtained with Cu13Ge after solution annealing and aging, reaching ~116 HV_0.1_.

**What are the implications of the main findings?**

- Cu13Ge is identified as the most promising filler for achieving mechanically reliable CuCrZr–W joints.
- Post-brazing heat treatments are essential to recover the mechanical properties of CuCrZr for fusion-relevant operating conditions.
- Optimizing the brazing process supports the development of DEMO divertor components by improving structural integrity and heat-dissipation performance.

**Abstract:**

This study investigates the brazeability of tungsten (W) and a CuCrZr alloy produced by means of additive manufacturing, using four different filler compositions from the Cu-Ge and Cu-Ti systems. The additive process resulted in a CuCrZr alloy with a columnar grain structure and a fine nanodispersion of Cr-rich strengthening precipitates. Brazing with W was performed using three Cu-Ge fillers: Cu13Ge, Cu19Ge, and Cu33Ge, at 1030, 900, and 775 °C, respectively. Increasing the Ge content reduced the brazing temperature but increased brittleness in the braze zone. Only with the highest Ge content (Cu33Ge) was a lack of metallic continuity at the interface observed. A fourth filler, Cu-20Ti, was used at 960 °C, but the braze zone exhibited cracks due to the presence of Ti-rich phases. The hardness of W remained unaffected after brazing. However, the CuCrZr alloy experienced softening caused by the loss of fine precipitate dispersion. To restore the required hardness for application, joints brazed with Cu13Ge and Cu19Ge—those with the best results—underwent post-brazing treatments including solution annealing, quenching, and aging. Cu13Ge joints showed optimal recovery with long annealing times (60 min), achieving a hardness of 116.2 ± 4.5 HV_0.1_ after aging for 120 min at 450 °C.

## 1. Introduction

The DEMO (DEMOnstration Power Plant) project is a European initiative aimed at developing a fusion reactor that promises to revolutionize the future of clean and sustainable energy generation. Building upon the extensive knowledge gained from experimental fusion reactors like ITER (International Thermonuclear Experimental Reactor), DEMO represents a critical step towards realizing practical fusion power. Its primary goals are to demonstrate the feasibility and commercial viability of large-scale fusion energy, produce substantial amounts of net electricity, and showcase the key technologies and engineering solutions required for future fusion power plants. Through its development and subsequent operation, DEMO seeks to address and overcome the numerous challenges associated with practical fusion power generation, thereby paving the way for a sustainable energy future [1].

The divertor is a critical component of a fusion reactor, designed to deal with the extreme heat and particle flux generated during fusion reactions. It acts as a “heat sink” to extract the high-energy particles and dissipate the generated heat, ensuring the stability and longevity of the reactor. One of the most promising designs for the divertor features a monoblock structure, which consists of tungsten monoblocks joined to a CuCrZr pipe [2].

The tungsten monoblock forms the primary surface of the divertor that directly interacts with the plasma. Tungsten is chosen for its exceptional high-temperature properties, including its high melting point, low erosion rate, and excellent thermal conductivity [3]. These properties allow this material to withstand intense heat and particle erosion in the divertor region [4,5]. The CuCrZr pipe is an essential part of the divertor assembly, providing structural support and heat transfer capabilities. This pipe is typically made of a copper–chromium–zirconium alloy, selected for its high thermal conductivity and mechanical strength at temperatures higher than pure Cu [6,7]. Acting as a coolant channel, the CuCrZr pipe carries a coolant, such as water, to absorb the excess heat generated by the plasma. Its high thermal conductivity allows efficient heat transfer from the tungsten monoblock to the coolant, preventing overheating and ensuring optimal divertor performance [8,9,10].

Additive Manufacturing, commonly known as 3D printing, has emerged as a revolutionary technology with the potential to transform multiple industries. Unlike traditional manufacturing methods that involve subtracting or molding materials, additive manufacturing creates objects by adding successive layers of material, enabling the production of complex and intricate designs. One of the most significant advantages of additive manufacturing is its ability to fabricate highly complex geometries that would be impossible or expensive to produce using traditional methods. The design freedom inherent in additive manufacturing fosters creativity and enables the production of lighter, stronger, and more efficient components [11,12].

This technology is being widely used by the scientific community, both for the design of parts with complex geometries and the in situ repair of plasma-facing materials. In the case of the CuCrZr alloy, which is studied in this paper, Nomura et al. [13] studied the production of Cu-alloy-based components using laser powder bed fusion to fabricate strong and ductile CuCrZr alloys. They prepared a CuCrZr alloy with high strength and ductility of 287 MPa and 42%, respectively, and effective precipitation strengthening. B. Liu et al. [14] used Selective Laser Melting (SLM) technology to prepare hybrid parts made of two materials by depositing CuCrZr on a 316L stainless steel substrate. They demonstrated that the microstructure of SLM-ed CuCrZr consisted of elongated columnar grains oriented along the building direction, while the 316L matrix material was characterized by large equiaxed grains. After polishing the 316L substrate, no macroscopic cracks were observed, and only a few pores were detected at the 316L/CuCrZr interface, indicating an overall good metallurgical bond. Hu et al. [15] studied the evolution of the microstructure and mechanical properties of CuCrZr manufactured by means of SLM with different scanning parameters. They showed that the CuCrZr specimens with high relative density (99.5 ± 0.3%) possessed the highest strength (280 ± 6 MPa) and ductility (23.4 ± 0.4%) compared to other SLM-manufactured specimens.

In order to ensure thermal transmission from tungsten to the CuCrZr pipe, a metallic bond is considered. Among the different bonding techniques, brazing has been widely used for centuries to create strong and durable connections between metal components. This process utilizes a heterogeneous filler material, known as a braze alloy or filler metal, to bond two or more metal pieces together at temperatures lower than the melting points of the base materials. It relies on the principle of capillary action to distribute the molten filler metal into the joint, creating a solid and reliable bond [16].

Several authors have studied the brazing process of W to CuCrZr. Peng et al. [17] studied brazed joints between W and CuCrZr and W and stainless steel (SS) using a copper base alloy Cu-22TiH2 for both samples. They demonstrated complete metallic continuity and achieved shear strengths of 96 ± 18 MPa for W/CuCrZr joints and 98 ± 21 MPa for W/SS301 joints, respectively, indicating strong bonding between the filler and the substrates. On the other hand, Singh et al. [18] studied the joint of multi-layered W/Cu-CuCrZr-SS316L-SS316 using vacuum brazing with NiCuMn37 as a filler. They found through non-destructive testing that there were no signs of debonding at the interface region of the W-Cu, Cu-CuCrZr, and CuCrZr-SS joints. Moreover, shear strength measurements of the multilayered brazed joints revealed that the Cu-CuCrZr joint interface exhibited a maximum shear strength of 175 MPa, whereas the W-Cu interface showed considerably lower strength values of 22 MPa.

It is necessary to combine all of the previously mentioned advantages, from additive manufacturing to the use of brazing as a joining technique. For this, it is necessary to optimize the additive manufacturing process of the samples as well as the correct choice of the filler material for brazing. Copper alloys have been widely utilized in nuclear applications and similar alloys have been studied within our group, obtaining positive results for CuTi [19] and CuGe alloys [20]. Therefore, comprehensive investigations were conducted to understand the effects of varying brazing temperature and dwell times, with results discussed based on the resulting microstructure in each case [21,22].

The objective of this study is to examine the brazeability of a CuCrZr alloy produced via additive manufacturing and investigate the impact of thermal treatments on the microstructure and properties of the alloy. Various thermal treatments were performed to restore the hardness of the as-received CuCrZr alloy. The brazing technique emerged as a suitable joining technique for these materials and applications and will be utilized in this research. Different copper alloys based on the Cu-Ti and Cu-Ge systems will be employed as fillers to join the base materials.

## 2. Materials and Methods

### 2.1. Materials and Fabrication of the Fillers

Powder with the composition listed in Table 1 was obtained at Ceit using gas atomization with a close-coupled gas atomization unit. The target composition was selected to meet ITER specifications and minimize the concentration of impurities such as Fe, Si, or O, which could affect the thermal conductivity of the alloy. The aluminum concentration was maintained below 200 ppm to prevent activation issues. The powders were sieved to select particles ranging from 45 to 106 µm, ensuring a particle size distribution suitable for Electron Beam Powder Bed Fusion (EB-PBF). Aidimme (San Sebastián, Spain) processed the resulting CuCrZr powder batch using an Arcam A2X EB-PBF system (Mölndal, Sweden). The fabrication process parameters followed are described in Table 2. Following EB-PBF, samples underwent Hot Isostatic Pressing (HIP) treatment at 450 °C and 150 MPa for 3 h to eliminate residual porosity that may remain in the as-built material while avoiding overaging (coarsening of Cr precipitates). This fabrication route was selected based on the study reported in [23], where parameters such as scanning speed, beam current, and line offset were optimized. The selected conditions have shown promising results, producing nearly fully densified components via EB-PBF. Thermal conductivity measurements obtained from the densified specimens are consistent with the required thermal performance criteria for High Heat Flux components.

Hot-rolled tungsten base material, supplied by Plansee, was used in the form of plates with a final thickness of 5 mm. It had a high purity level of 99.99% and recrystallized microstructure associated with the hot rolling process.

Various filler compositions were tested for joining W to CuCrZr base materials, and their suitability and brazeability were studied. These compositions include three from the Cu-Ge system, Cu13Ge, Cu19Ge, and Cu33Ge (wt.%), and one from the Cu-Ti system, Cu20Ti (wt.%). Copper, titanium, and germanium metallic powders were provided by Cymit Química, with copper powders available in −325 mesh, +325 mesh, >99% purity; titanium powders in −200 mesh, 99.5% purity; and germanium powders in −100 mesh, 99.999% purity.

Considering the operating range of 500–600 °C [24], the brazing temperature window should be positioned above the service temperature and below the melting point of the Cu-based alloy (1085 °C) [24]. The proposed Cu–Ge and Cu–Ti filler compositions meet these criteria, making it essential to evaluate their brazing performance.

Tape fillers of 150 μm thickness, obtained from a master alloy of each composition, were used as filler materials. Master ingot alloys were obtained from a mixture of metallic powders with the selected compositions. These mixtures were compacted under 750 MPa pressure for 2 min to produce pellets of 13 mm in diameter. The pellets were then melted at 100 °C above the melting range of each alloy for 1 h in a high vacuum furnace, which allows working under vacuum with a residual pressure of 10^−6^ mbar. Finally, thin fillers were obtained by cutting slices from the ingot and grinding them to the final thickness.

### 2.2. Brazing Process and Post-Brazing Heat Treatments

Brazing tests were conducted using a high vacuum furnace (Navertherm, Lilienthal, Germany) with a residual pressure of 10^−6^ mbar. The base materials’ dimensions were copper alloy and tungsten blocks with an exposed surface of 6 × 6 mm2 and 4 mm thickness. Before the brazing tests, the exposed surfaces of both base materials were polished using 4000-grit silicon carbide paper.

Brazing temperatures were selected according to each alloy’s melting range, applying temperatures 50 degrees above the liquidus temperatures, which were determined in previous studies (Table 3) [20,21].

The brazing cycle consisted of heating up to the brazing temperature, holding that temperature for 10 min, followed by cooling to room temperature. The heating and cooling rates were maintained at 5 °C/min.

Following the evaluation of brazing results, suitable heat treatments were studied to recover the base material properties. These treatments aimed to regenerate the initial microstructure, especially the dispersion of the strengthened precipitates, which confers the superior mechanical properties of this alloy in comparison with pure Cu. A three-stage process was applied for that purpose, comprising solution annealing, quenching, and aging treatments.

According to experimental characterization, Cu13Ge and Cu19Ge have solidus temperatures of 925 °C and 800 °C, respectively. Hence, 25 °C lower solution annealing temperatures were chosen to achieve the maximum degree of solubilization without causing remelting of the braze. Annealing temperatures of 900 °C and 775 °C were chosen for Cu13Ge and Cu19 Ge filler compositions, respectively. Two dwell times were analyzed to assess their effect on alloying solubility: 30 and 60 min for 900 °C annealing temperature, and 60 and 120 min for 775 °C. Water at room temperature was used as the quenching agent. Finally, two different aging temperatures were also analyzed: 450 °C and 500 °C, applied for 0, 15, 60, 120 and 180 min.

The evaluated conditions are listed in Table 4.

### 2.3. Characterization Techniques

The metallographic examination of the specimens involved cross-sections cut using a standard polishing technique for surface preparation. Scanning electron microscopy (SEM, S3400 Hitachi, Tokyo, Japan) equipped with Energy Dispersive X-ray Spectroscopy (EDS) analysis was employed for this purpose. Precipitate distribution and size analysis of the CuCrZr base material were performed using a stereoscopic microscope (Leica DMR, Weztlan, Germany) with Leica Image Pro Plus software (Leica Application Suite Version 4.8.0 (build: 154)) and an Optical Microscope (OM). Furthermore, the scanning transmission electron microscopy technique (STEM, JEOL F200, Akishima, Japan and Zeiss Sigma 500, Oberkochen, Germany, equipped with a GEMINI column, Tokyo, Japan) was also used to examine the nanostructure of the Cu alloy. For this, focused ion beam equipment (Leica RES102, Weztlan, Germany) was used to prepare the samples for TEM examination. Some samples were etched before microscopic observation to enhance the microstructure examination; a solution of 5 g FeCl_3_, 2 mL HCl, and 95 mL ethanol was used for etching CuCrZr and the braze zone.

The potential impact of the brazing thermal cycle on the base materials’ hardness was determined through Vickers microhardness measurements. Microhardness profiles from the CuCrZr side to the tungsten side across the braze were obtained using an MHV-2SHIMADZU instrument (Dallas, TX, USA). A 100 g load was applied for 15 s. To ensure the repeatability of the measurements, three indentations were made per distance, and the mean value along with its standard deviation was used to discuss the results. The separation between neighboring indentations was maintained at a distance greater than three times the residual imprint sizes.

## 3. Results

### 3.1. Characterization of CuCrZr Base Material

Considering the innovative nature of the CuCrZr alloy fabrication route, the copper alloy produced via electron beam melting additive manufacturing was subjected to microstructural and mechanical characterization before its joining with tungsten.

**Figure 1:**
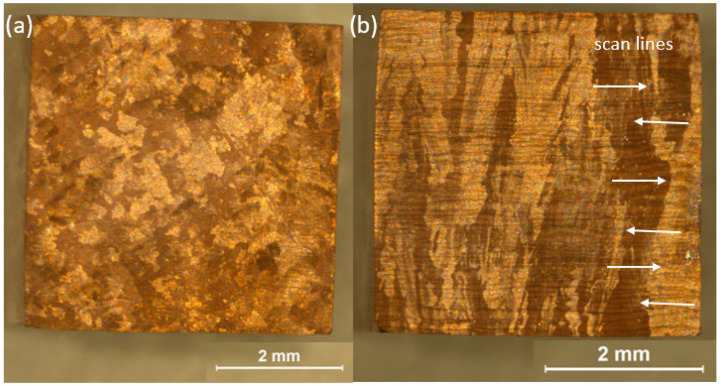
Micrographs of the CuCrZr alloy obtained using a stereoscopic microscope after chemical etching: (**a**) transverse direction and (**b**) longitudinal direction.

Figure 1a and Figure 1b present the etched microstructure of the transverse and longitudinal planes, respectively, relative to the sample building direction, as observed with a stereoscopic microscope. The longitudinal view reveals a columnar grain structure aligned with the building direction, a typical feature of this manufacturing process. The average grain size is 670 ± 150 µm in width and 2440 ± 470 µm in length, with the scan pattern from the fabrication process distinctly visible. In contrast, the transverse view displays equiaxed grains with an approximate size of 420 ± 280 µm, highlighting the anisotropic nature of the microstructure.

**Figure 2:**
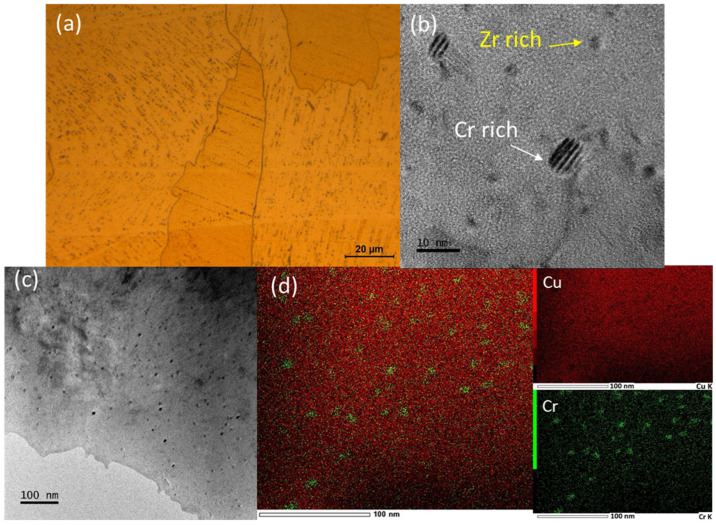
Analysis of precipitates in the CuCrZr base material: (**a**) Optical microscopy, (**b**) high-resolution TEM, (**c**) TEM, and (**d**) elemental mapping distribution.

A detailed analysis of the base material microstructure is presented in Figure 2. Optical, FEG-SEM, and TEM microscopy were used for microstructural characterization. As shown in Figure 2a, a finely dispersed and homogeneously distributed population of precipitates is observed, primarily located within the grains. These precipitates result from the segregation and precipitation of Cr along the boundaries of the columnar submicrometric grains during the EB-PBF process, which occurs at a powder bed temperature of approximately 380 °C. The formation of these columnar structures is characteristic of additive manufacturing techniques with high cooling rates and strong thermal gradients. Additionally, substructure tilting is observed when the scan pattern rotates by 90°, as implemented in this study [23,24].

FEG-SEM analysis (Figure 2b) revealed not only the distribution of fine precipitates but also the presence of coarser ones. These larger precipitates display a more heterogeneous distribution, appearing both within the grains and along grain boundaries. Semi-quantitative EDS analysis identified the fine precipitates as Cr-rich, while the coarser ones were Zr-rich.

Additionally, TEM examination confirmed the homogeneous distribution of fine precipitates, with particle sizes ranging from 2 to 5 nm (Figure 2b). The STEM elemental distribution map further emphasizes the Cr-rich nature of these precipitates (Figure 2d).

Mechanical characterization was performed through hardness measurements, yielding an as-received hardness of 222 ± 5 HV_0.1_. Typically, the fine dispersion of hardening precipitates forms through solution annealing, quenching, and subsequent aging. The inherent nature of the fabrication process, involving melting and rapid cooling, effectively achieves the first two stages. The aging stage was carried out at 450 °C and 150 MPa for 3 h, as described in the experimental procedure.

Unlike conventional fabrication routes, precipitation hardening in this case is driven by nanometric precipitates, leading to a greater hardening effect on the alloy’s final properties. Additionally, the high cooling rates inherent to the manufacturing process induce further hardening due to the generation of residual stresses. These factors account for the higher hardness of the alloy compared to conventional processing methods, which typically yield values of approximately 166 HV_0.1_ for forging or hot rolling [25] and 135 HV_0.1_ for hydrostatic extrusion and equal-channel angular pressing [26,27].

**Figure 3:**
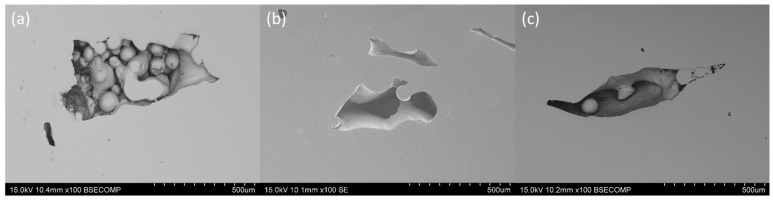
Different defects in the CuCrZr base material of the as-received samples: (**a**) Cu13Ge, (**b**) Cu19Ge, and (**c**) Cu20Ti.

It is important to highlight the porosity present in isolated areas of the CuCrZr base material, as shown in Figure 3a–c. This porosity corresponds to typical defects associated with the production process, such as a lack of fusion, where unmelted particles of the copper alloy are observed.

### 3.2. Microstructural Characterization of Brazed Joints

Four different filler compositions were selected for brazing W to CuCrZr, based on the Cu-Ge and Cu-Ti systems [28], respectively. For the Cu-Ge system, the melting range decreases progressively with increasing Ge content. Three compositions were chosen—Cu13Ge, Cu19Ge, and Cu33Ge—to evaluate the effect of varying brazing temperatures on the CuCrZr base material. Furthermore, the potential for restoring the as-received properties after the joining process through various post-brazing thermal treatments was also investigated.

The effect of Ge content on the microstructure and mechanical properties of the braze was analyzed. The first two compositions were influenced by peritectic reactions, while the third was impacted by both peritectic and eutectic reactions. For the Cu-Ti system, the selected composition was influenced by a eutectic reaction and corresponds to the lowest melting temperature in the system, aimed at controlling the thermal effect.

Moreover, when selecting a brazing procedure, it is advantageous to choose brazing fillers that closely match the eutectic composition. This reduces the melting range, and the closer the composition is to the eutectic point, the better the flow properties. Eutectic compositions typically exhibit excellent fluidity, as the solid and liquid phases do not coexist during the heating stage. This facilitates capillary action, helps retain the liquid, and effectively fills the joint clearance.

#### 3.2.1. Cu13Ge

**Figure 4:**
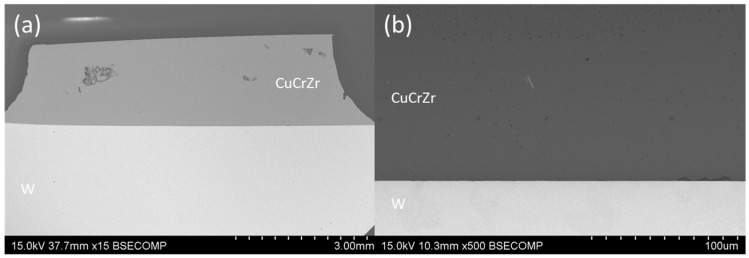
SEM micrographs of the W-CuCrZr joint brazed with Cu13Ge filler: (**a**) General view and (**b**) detail of the brazed area.

Brazing with the lowest Ge filler content was performed at 1030 °C. Figure 4a shows a general view of the braze joint, where the isolated porosity previously detected in the CuCrZr alloy does not affect the brazed area. The backscattering images in Figure 4b confirm the achievement of full metallic continuity at both interfaces. No distinct phases were observed in the brazed area, as the atomic weights of the phases are similar, resulting in no contrast when using this detector. Therefore, etching of the sample is necessary to reveal the microstructure.

**Figure 5:**
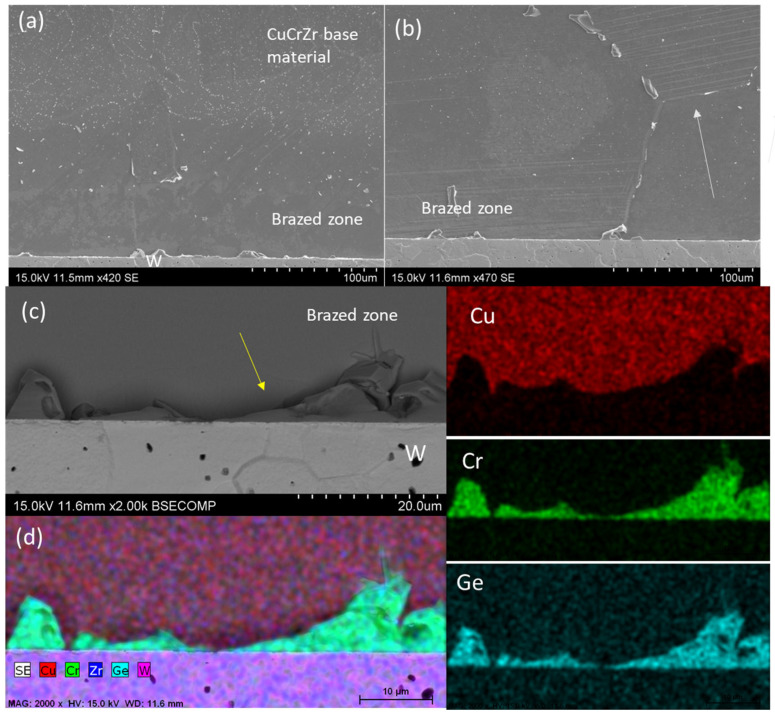
SEM micrographs after etching of the joint W-CuCrZr with Cu13Ge: (**a**) General overview, (**b**) detail of the CuCrZr interface, (**c**) detail of the W interface, and (**d**) EDS of the Cr-Ge rich phase in the W-braze interface.

The micrographs obtained after etching (Figure 5) clearly reveal the braze seam, which is characterized by the absence of the fine dispersion of precipitates typically observed. In contrast, this fine dispersion is prominently visible in the CuCrZr base material. Within the braze, several distinct phases can be identified, which are associated with the interaction between the braze and the CuCrZr base material. However, under these conditions, tungsten does not undergo metallurgical interaction with the braze due to its inert nature at the temperatures involved.

The braze is primarily composed of a copper matrix phase, as confirmed by the EDS point analyses and the elemental distribution map shown in Figure 5d. This suggests that the braze will predominantly exhibit ductile behavior, enabling residual stresses to be partially relieved through plastic deformation mechanisms during operation. As a result, the likelihood of brittle fracture is reduced, which is particularly important due to the mismatch in the CTE of the base materials (4.5·10^−6^ K^−1^ for tungsten [20] and 16.6·10^−6^ K^−1^ for CuCrZr [29]).

The second phase present is a Ge- and Cr-rich phase, with a composition of 63Cr30Ge5Cu1Zr at.% (indicated by the arrow in Figure 5c). This suggests that during the brazing process, chromium from the CuCrZr alloy near the braze has diffused or been diluted into the braze, forming the Cr-Ge-rich phase upon solidification. This phase preferentially solidified at the W-braze interface (yellow arrow in Figure 5c) and along the grain boundaries (Figure 5b).

Additionally, due to the cooling rate, martensitic transformations are observed at the interface (Figure 5b, white arrows).

#### 3.2.2. Cu19Ge

**Figure 6:**
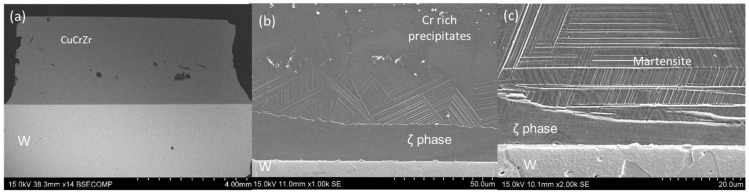
SEM micrographs of the W-CuCrZr joint brazed with Cu19Ge filler: (**a**) General view of the joint after brazing, (**b**) detail of the braze after etching, and (**c**) detail of the martensite at the W-braze interface.

The use of the intermediate Ge content filler resulted, as in the previous case, in high metallic continuity throughout the interfaces. No porosity or wettability defects are observed in the braze area (Figure 6a).

After etching, the microstructure reveals the presence of two main phases (Figure 6b,c). The phase in contact with the CuCrZr base material has an 88Cu10Ge1Cr1Zr at.% composition. At the bottom of this phase, a martensitic microstructure was formed (Figure 6c). Additionally, at the interface with CuCrZr, Cr diffused into the filler and precipitated (Figure 6b). The second phase, located at the W interface, was formed by a peritectic reaction. This phase is richer in Ge content and has an 85Cu13Ge1Cr1Zr at.% composition, which likely corresponds to the ζ phase of the phase diagram.

As expected, the lower temperature used in this case (900 °C) with respect to the first composition did not produce interaction of the braze alloy with the W base material.

#### 3.2.3. Cu33Ge

**Figure 7:**
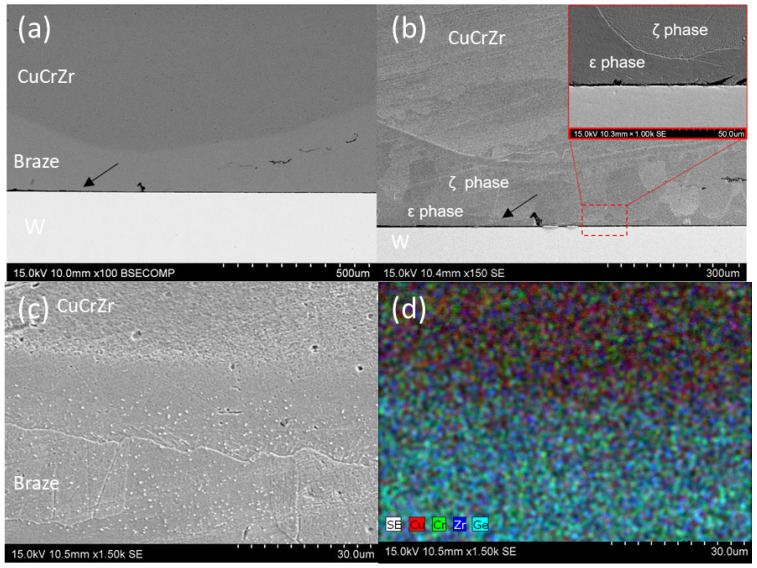
SEM micrographs of the W-CuCrZr joint brazed with Cu33Ge filler: (**a**) General view of the joint (**a**) before and (**b**) after etching. (**c**) Detail of the interface with the CuCrZr base material. (**d**) Elemental mapping distribution of (**c**). (black arrow in (**a**,**b**) show defects of the joint).

W-CuCrZr joints using Cu33Ge filler exhibited reduced metallic continuity at the W-braze interface. This effect could be attributed to the poor wetting behavior of the braze on tungsten or to the stresses generated during the cooling stage, due to the mismatch in the CTE between the base materials, leading to interface detachment (indicated by the black arrow in Figure 7a). Several studies have shown that the residual stress accumulated at this interface in such joints can reduce the brazeability, especially if the braze is not ductile enough due to the increased Ge content [30,31,32].

Microstructural analysis after etching revealed that the interaction front at the CuCrZr-braze interface extends into certain areas of the CuCrZr base material, resulting in a rounded interface typical of liquid–solid interactions (Figure 7b). Based on the EDS analysis, the microstructure appears to consist of the ζ phase (80Cu17Ge1Cr2Zr at.%) in contact with the CuCrZr base material, a ζ matrix with ε needles (Figure 7c), and the ε phase (73Cu25Ge1Cr1Zr at.%) at the W-braze interface. Figure 7d illustrates the graded distribution of elements at the CuCrZr interface, resulting in a less pronounced interface compared to previous cases.

#### 3.2.4. Cu20Ti

**Figure 8:**
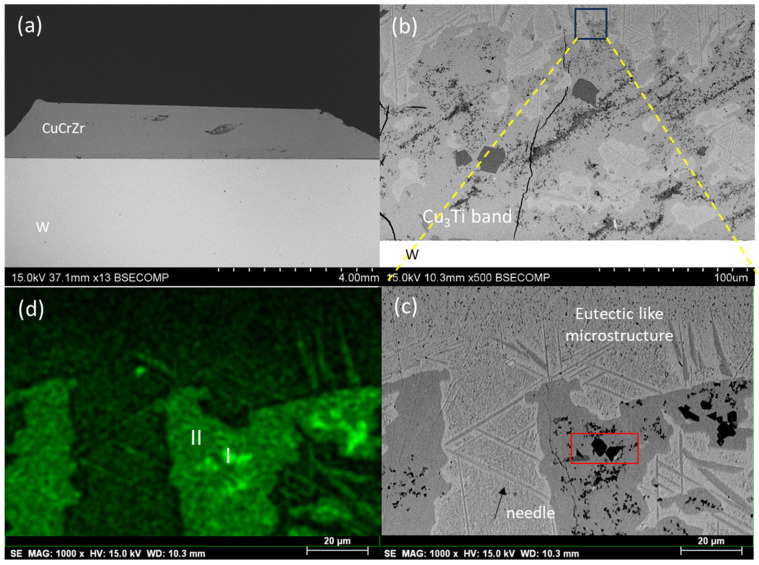
SEM micrographs of the joint W-CuCrZr brazed with Cu20Ti filler: (**a**) General view of the joint, (**b**) detail of defects, (**c**) close-up of the area marked in (**b**) with the presence of needles marked with black arrow; and (**d**) elemental mapping distribution of Ti of (**c**) with Cu3Ti band and Ti precipitates marked as I and II.

The last filler composition studied was Cu20Ti, brazed at 960 °C. Figure 8a shows the resulting microstructure after the brazing process. In this case, a significantly different microstructure compared to the Cu-Ge system was observed, characterized by the presence of various phases in the form of bands and precipitates of different sizes. These conditions resulted in high metallic continuity; however, some defects, such as cracks, appeared in the braze area due to the brittle nature of certain phases (Figure 8b).

**Figure 9:**
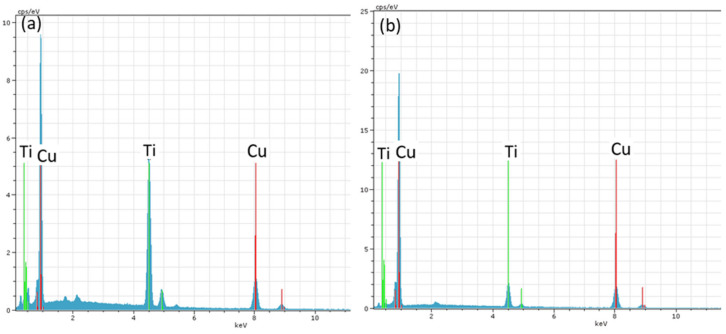
EDS spectra of phases I (**a**) and II (**b**) in Figure 8c.

The braze is characterized by two main phases. The first phase, primarily located as a band at the W interface, has a Cu-Ti composition according to the EDS results. Previous studies [19] suggest that this phase corresponds to the crystalline Cu3Ti phase (Figure 8b). This phase penetrates into the braze area at certain points. The high magnification image of the Cu3Ti band in Figure 8d reveals the presence of titanium-rich precipitates as black dots (53Ti47Cu at.%) within this phase. The second phase, which appears as a light contrast in the backscattering image (Figure 8b), consists of Cu3Ti needles (indicated by the black arrow in Figure 8c) and a eutectic microstructure of Cu3Ti and Cu (Figure 8c). Figure 8d shows the Ti distribution of the phases shown in Figure 9, with EDS spectra of the Cu3Ti band and Ti precipitates marked as I and II, respectively.

As in the other cases, no interaction was observed between the braze or the CuCrZr base material and the W.

### 3.3. Mechanical Characterization of Brazed Joints

**Figure 10:**
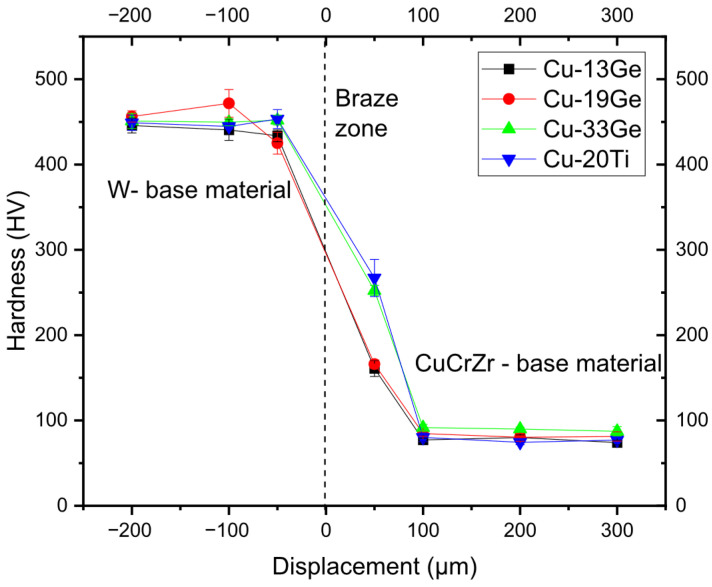
Microhardness profiles of the W-CuCrZr brazed joint across the braze.

The mechanical characterization of brazed joints was performed through microhardness testing. Figure 10 presents the microhardness profile of W-CuCrZr joints brazed with the selected Cu-Ge and Cu-Ti filler compositions. The results indicate that the hardness of tungsten remained unaffected by the different brazing cycles, maintaining a value close to 450 HV_0.1_. The average hardness across all conditions falls within the error range compared to reported literature values for polycrystalline tungsten in its as-received state [33]. These findings align with the microstructural analysis, which showed no metallurgical interaction of tungsten at the interface, and the applied temperatures remained below those required to induce recrystallization [34].

In the case of the CuCrZr base material, the brazing conditions induce a softening process. In all cases, hardness values below 100 HV_0.1_ were measured, which are significantly lower than those observed in the as-received condition (222 ± 5 HV_0.1_). This softening can be attributed to several mechanisms, including grain growth at high temperatures, but primarily to the partial solubilization or depletion of hardening precipitates. Although the brazing process typically involves short dwell times, especially compared to other heat treatment techniques, the elevated temperatures can still initiate the solubilization of precipitates, particularly in processes with higher brazing temperatures. This explanation is consistent with the experimental results, where increased brazing temperatures led to greater reductions in base material hardness. For instance, average hardness values of 75 ± 4 HV_0.1_ and 93 ± 4 HV_0.1_ were recorded for Cu13Ge and Cu33Ge, respectively, which were brazed at 1030 °C and 775 °C.

**Figure 11:**
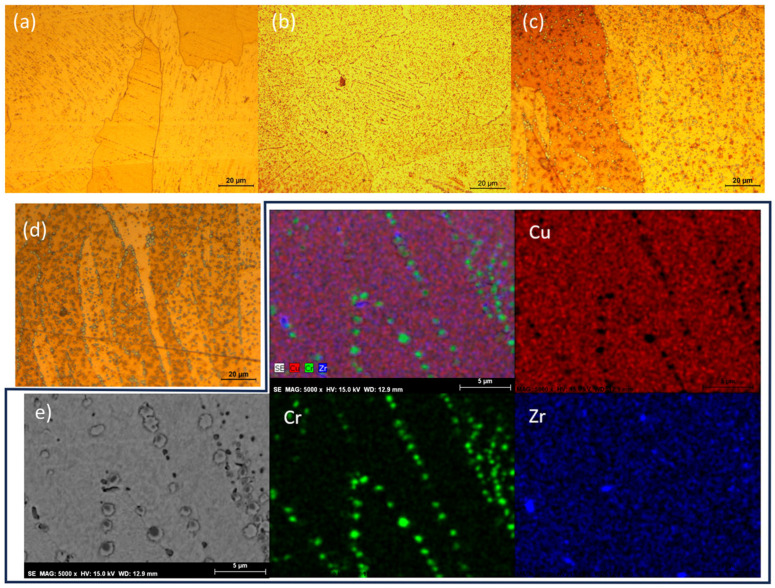
Optical micrographs of CuCrZr: (**a**) as-received condition, (**b**) after brazing at 900 °C, (**c**) after brazing at 1030 °C, and (**d**) detailed view of precipitates from (**b**,**e**) elemental mapping distribution for the sample brazed at 1030 °C.

Based on the results, a detailed analysis of the precipitate distribution in the CuCrZr base material was conducted for each condition. Figure 11a shows the precipitate distribution in the as-received condition, where a fine and homogeneous dispersion of hardening precipitates is observed. However, after the brazing process, the micrographs of the base material (Figure 11b and Figure 11c, corresponding to brazing temperatures of 900 °C and 1030 °C, respectively) reveal that the precipitates have grown and preferentially accumulated along the grain boundaries. Additionally, at higher brazing temperatures, the precipitates appear larger than those formed at lower temperatures.

During brazing, the temperatures reached placed the material within the solution annealing field, as indicated by the equilibrium phase diagram [28]. However, only a fraction of the precipitates dissolved (0.57 wt.% at 1030 °C and 0.28 wt.% at 900 °C), while the remaining precipitates underwent a coarsening process. During cooling, the reduction in solubility led to the precipitation of alloying elements, predominantly along the grain boundaries, shaping the final microstructure. This phenomenon explains the hardness test results. The EDS distribution map in Figure 11d confirms that most grain boundary precipitates are chromium-based, with only a few isolated Zr precipitates also observed at the grain boundaries.

The observed softening process could impact the mechanical performance of the CuCrZr alloy in its application within the fusion reactor. According to ITER specifications, this alloy must maintain a minimum hardness of 124 HV_0.1_ to ensure reliable mechanical performance at both room and high temperatures [35]. Therefore, it is essential to explore methods for restoring or achieving this required hardness. In this study, samples brazed at 900 °C and 1030 °C, identified as the conditions that provided the best brazeability, underwent a post-brazing treatment consisting of solution annealing, quenching, and aging to restore the microstructure and hardness of the CuCrZr alloy.

**Figure 12:**
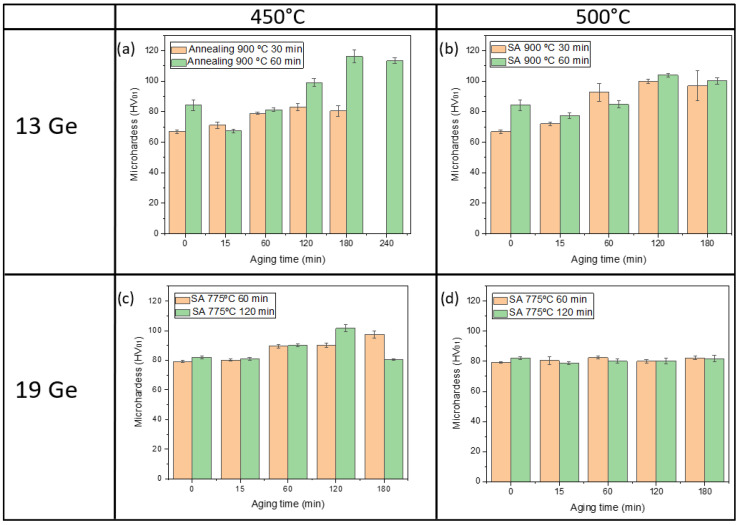
Microhardness of CuCrZr base material after undergoing aging treatment. Using Cu13Ge filler, aging at (**a**) 450 °C and (**b**) 500 °C; using Cu19Ge filler, aging at (**c**) 450 °C and (**d**) 500 °C.

The results of the various post-brazing treatments are presented in Figure 12, where a typical hardening curve associated with precipitation strengthening is observed.

Rapid cooling (quenching) after solution annealing results in a slight increase in hardness compared to the values obtained after brazing. This increase can be attributed to solid solution hardening [36]. During the subsequent aging treatment, the supersaturated solid solution begins to decompose, leading to the formation of Zr- and Cr-rich precipitates, which contribute to hardening, as previously discussed. A study by D. Liu et al. [24] demonstrated that the precipitation of hardening phases in CuCrZr alloys follows a sequential mechanism, beginning with the formation of Cr-rich phases and Cu-Zr composite precipitates. According to the study, Cr and Zr precipitates undergo morphological transformations, shifting from lamellar to granular structures due to atomic interactions, which significantly enhance the material strength.

However, once peak strength is reached, hardness begins to decline, as observed in Figure 12. This overaging effect occurs due to the coarsening of the fine precipitate dispersion, reducing its ability to hinder dislocation movement, thereby leading to a loss in hardness.

The Cu13Ge filler exhibits a continuous increase in hardness as aging time progresses. At an aging temperature of 450 °C, significant differences are observed when varying the dwell time during solution annealing, particularly at longer aging durations. This suggests that prolonged solution annealing is necessary at this temperature to fully dissolve the alloying elements, initially present as precipitates, into solid solution. The maximum hardness achieved is 83.1 ± 2.3 HV_0.1_ and 116.2 ± 4.5 HV_0.1_ for solution annealing times of 30 and 60 min, respectively. Overaging effects are not observed until 240 min in samples subjected to 60 min of solution annealing. Increasing the aging temperature to 500 °C results in a less pronounced strengthening effect, with maximum hardness values of 99.8 ± 1.3 HV_0.1_ and 104.1 ± 1.1 HV_0.1_ for 30 and 60 min dwell times, respectively, both achieved after 120 min of aging.

For Cu19Ge filler joints, longer solution annealing times were selected to compensate for the lower diffusion coefficient at this temperature. In general, these joints exhibit lower hardness values compared to those using the Cu13Ge filler. This reduction is attributed to the lower solution annealing temperature, which limits the dissolution of alloying elements into the solid solution, as indicated by the equilibrium phase diagram [28]. Consequently, fewer hardening precipitates are formed during the aging process. At an aging temperature of 450 °C, a maximum hardness of 101.5 ± 1.5 HV_0.1_ is achieved after 120 min of solution annealing and aging. Lower hardness values were obtained for shorter annealing and aging durations. Finally, increasing the aging temperature to 500 °C does not result in any noticeable strengthening. This could be attributed to two factors: the reduced effectiveness of the strengthening process at higher aging temperatures and the shorter solution annealing time at this temperature, which further limits hardening potential.

## 4. Conclusions

This study explores the brazeability of a W to CuCrZr alloy produced via additive manufacturing. The as-received characterization of the CuCrZr alloy reveals a columnar grain structure typical of this manufacturing technique, with the presence of Cr-strengthening nanoprecipitates. Consequently, the material exhibits higher hardness (222 ± 5 HV_0.1_) compared to conventionally manufactured CuCrZr alloys.

The results demonstrate that brazing W to this CuCrZr alloy using Cu13Ge, Cu19Ge, and Cu33Ge fillers generally produces high-quality joints. However, at higher Ge concentrations (Cu33Ge), cracks and discontinuities were observed, highlighting a trade-off between reducing brazing temperature and increasing brittleness in the braze zone. The resulting microstructure is characterized by a homogeneous braze, where Cu, ζ, and ε phases are identified, forming well-defined compositional layers without secondary phases or precipitates.

In contrast, joints brazed with Cu-Ti fillers exhibit a more complex microstructure, consisting mainly of Cu and Cu3Ti phases with Ti-rich precipitates and needle-like structures. The presence of these Ti-rich phases contributes to increased brittleness, leading to crack formation during the cooling stage of the brazing process.

Mechanical characterization confirms that the hardness of W remains unaffected by any of the brazing processes. However, the CuCrZr alloy undergoes a softening process due to the loss of the fine dispersion of strengthening precipitates.

To restore the required hardness specified for ITER applications, joints brazed with Cu13Ge and Cu19Ge, identified as the most promising filler compositions in terms of brazeability, were subjected to post-brazing heat treatments, including solution annealing, quenching, and thermal aging. These treatments aim to reestablish the fine dispersion of strengthening precipitates.

For Cu13Ge joints, the best results were achieved with longer solution annealing times (60 min), reaching a maximum hardness of 116.2 ± 4.5 HV_0.1_ after 120 min of aging at 450 °C. A reduction in solution annealing time or an increase in aging temperature resulted in lower hardness values.

Joints brazed with Cu19Ge exhibited lower hardness values due to the lower solution annealing temperature, which limited the dissolution of alloying elements into the solid solution, thereby reducing the precipitation hardening effect.

Overall, this study demonstrates that Cu13Ge and Cu19Ge are promising filler materials for brazing W to CuCrZr, with post-brazing heat treatments proving effective in restoring the alloy mechanical properties. Further optimization of these treatments could enhance joint performance for high-temperature applications such as those in ITER.

## Figures and Tables

**Table 1:** Composition of CuCrZr powder elements.

Composition [wt.%]			Impurities [ppm]				
Cu	Cr	Zr	Fe	Si	Al	C	O
Bal.	0.95	0.067	110	120	<200	19	80

**Table 2:** EB-PBF fabrication process parameters.

Beam Current (mA)	8.5
Scanning Speed (mm/s)	170
Line offset (mm)	0.15
Focus offset (mA)	22
Layer thickness (µm)	70
Rotation angle between layers (°)	90
Powder bed temperature (°C)	>380

**Table 3:** Brazing temperatures used for each filler alloy.

Sample	Brazing Temperature (°C)
Cu13Ge	1030
Cu19Ge	900
Cu33Ge	775
Cu20Ti	960

**Table 4:** Aging process conditions applied to restore the properties of the CuCrZr base material (in parentheses, the treatment used to cool).

	Brazing		Solution Annealing (Quenching)		Aging (Air Cooling)	
Sample	T (°C)	t (min)	T (°C)	t (min)	T (°C)	t (min)
Cu13Ge	1030	10	900	30–60	450–500	15-60-120-180
Cu19Ge	900	10	775	60–120	450–500	15-60-120-180

## Data Availability

The original contributions presented in this study are included in the article. Further inquiries can be directed to the corresponding author.
